# The short-term effects of air pollution exposure on preterm births in Chongqing, China: 2015–2020

**DOI:** 10.1007/s11356-023-25624-2

**Published:** 2023-02-22

**Authors:** Xin Ming, Ziyi He, Yannan Li, Yaqiong Hu, Yunping Yang, Hongyan Chen, Qin Chen, Huan Yang, Wenzheng Zhou

**Affiliations:** 1 Women and Children’s Hospital of Chongqing Medical University (Chongqing Health Center for Women and Children), Longshan Road 120, Chongqing, 401147 China; 2 Institute of Toxicology, College of Preventive Medicine, Army Medical University (Third Military Medical University), Chongqing, 400038 China

**Keywords:** Preterm birth, Air pollution, Distributed lag non-linear models, Lag effect

## Abstract

Accumulating evidence suggested that the risk of preterm births (PTBs) following prenatal exposure to air pollution was inconclusive. The aim of this study is to investigate the relationship between air pollution exposure in the days before delivery and PTB and assess the threshold effect of short-term prenatal exposure to air pollution on PTB. This study collected data including meteorological factors, air pollutants, and information in Birth Certificate System from 9 districts during 2015–2020 in Chongqing, China. Generalized additive models (GAMs) with the distributed lag non-linear models were conducted to assess the acute impact of air pollutants on the daily counts of PTB, after controlling for potential confounding factors. We observed that PM_2.5_ was related to increased occurrence of PTB on lag 0–3 and lag 10–21 days, with the strongest on the first day (RR = 1.017, 95%CI: 1.000–1.034) and then decreasing. The thresholds of PM_2.5_ for lag 1–7 and 1–30 days were 100 μg/m^3^ and 50 μg/m^3^, respectively. The lag effect of PM_10_ on PTB was very similar to that of PM_2.5_. In addition, the lagged and cumulative exposure of SO_2_ and NO_2_ was also associated with the increased risk of PTB. The lag relative risk and cumulative relative risk of CO exposure were the strongest, with a maximum RR at lag 0 (RR = 1.044, 95%CI: 1.018, 1.069). Importantly, the exposure–response curve of CO showed that RR increased rapidly when the concentration exceeded 1000 μg/m^3^. This study indicated significant associations between air pollution and PTB. The relative risk decreases with day lag, while the cumulative effect increases. Thus, pregnant women should understand the risk of air pollution and try to avoid high concentration exposure.

**Supplementary Information:**

The online version contains supplementary material available at 10.1007/s11356-023-25624-2.

## Introduction

Preterm birth (PTB) is a serious complication of pregnancy and is used as a predictor of neonatal mortality (Rocha et al. 2022). Due to the imperfect development of various body systems, premature infants are prone to severe multiple systems diseases, and high mortality and disability (Hamilton et al. 2015; McCormick et al. 2011). In the past 20 years, the incidence of PTB is increasing worldwide, making PTB a global problem in public health. According to the reports, more than one in ten babies are born prematurely each year worldwide. In China, the PTB rate ranges from 5% to 15%, the second highest in the world, with more than 1.17 million premature babies born each year (Lim et al. 2012; Zhao et al. 2015).

Previous epidemiologic researches have suggested that PTB is the outcome of the combined action of multiple factors, such as life behavior, psychological behavior, and genetic factors (Falah et al. 2013). Although there is such a growing body of studies assessing the influence of air pollutant exposure on PTB, some of these studies only focus on a certain single pollutant or use a sample size of PTB that is relatively limited. Further still, the results of the correlation between air pollution and PTB are inconsistent and not suitable to directly extrapolate them to areas with higher levels of air pollution. Moreover, the environmental air pollution components and concentrations in these studies have also varied (Li et al. 2021a, 2021b; Siddika et al. 2020; Smith et al. 2020; Warren et al. 2020). In summary, therefore, in rapidly developing countries with high concentrations of air pollutants, extremely high levels of air pollutants may increase the risk of PTB (Li et al. 2018; Qian et al. 2016).

However, the research on acute effects and analysis of air pollutants exposure on PTB has been less reported. It has also been hypothesized that the short-term relationship between PTB and air pollutants might relatively difficult to detect because of the seasonal character of PTB rates (Darrow et al. 2009; Stieb et al. 2019).

The goal of this paper, therefore, was to assess the influence of acute maternal exposure to air pollution on PTB in Chongqing, China, for the years 2015–2020. It is also meaningful to explore the thresholds for the risk of PTB due to short-term prenatal air pollution exposure, and thereby improve the understanding of PTB prevention overall.

## Material and methods

### Study area

This study was conducted in Chongqing, China, using 6-year daily data from a retrospective cohort study with multicenter. Chongqing has an area of 82,400 km^2^ and is located between 105°11′ N and 28°11′ E. It is an iron, petrochemical and aluminum industrial center in the southwest of China. Chongqing is known as the “Fog City” because of its special basin topography and meteorological conditions that impede the diffusion of ambient air pollutants. According to data gathered from the Chongqing Municipal Bureau of Statistics in 2020, Chongqing has a total population of 32.05 million. Its urban population accounts for 65.9% of that total, which ensures the stability of the population characteristics in this research effort. The main urban area had closely connected districts: Yuzhong, Jiangbei, Dadukou, Shapingba, Jiulongpo, Nanan, Beibei, Yubei, and Banan.

### Data collection

In this study, we obtained the birth outcome data from the Chongqing Birth Certificate System, collected between 2015 and 2020. The birth certificate data includes the child’s date of birth, permanent address, gestational age, etc. After childbirth, birth information of the baby is filled by health care attendants or midwives in the Neonatal Care Record System, which is then would be verified logically and specifically and uploaded to the information system. Before the birth certificate is issued, the parents and the Healthcare Commission confirm the information. We excluded those data that lacked gestational age and non-urban residents. Our analyses were based on 59,8018 births after exclusions, and a total of 35,044 premature babies were included. PTB was defined as a birth with less than 37 weeks of gestational (Blencowe et al. 2012; Warren et al. 2020). The birth certificate data was used to evaluate the number of PTB babies in Chongqing during the designated research period.

### Exposure assessment

During the study period, ambient air pollutant concentrations were obtained from the Chinese National Urban Air Quality Monitoring Platform (http://zhb.gov.cn) for 17 ground-based monitoring stations in Chongqing. We calculated the 24-h average particles with a diameter < 2.5 and < 10 μm (PM_2.5_, PM_10_), nitrogen dioxide (NO_2_), sulfur dioxide (SO_2_), and carbon monoxide (CO); ozone (O_3_) was an 8 h maximum value concentration. For each day, we averaged the data from the available monitors to compute mean values for the whole city. Daily average relative humidity and temperature were available from the China Greenhouse Data Sharing Platform (http://data.sheshiyuanyi.com). Imputation of missing data was done using multiple linear interpolation based on other monitors’ values.

### Statistical analysis

We used a quasi-Poisson GAM with the distributed lag non-linear models (DLNMs) to estimate associations of daily new case of PTB with air pollution exposure. The model was the following:

$$Log[E\left({Y}_{t}\right)]=\alpha +cb\left({Pollution}_{t},lag\right)+cb\left({Temp}_{t},lag\right)+cb\left({RH}_{t},lag\right)+ns\left({Time}_{t},df\right)+as.factor\left({DOW}_{t}\right)+as.factor({holiday}_{t})$$

where $t$ as the observation day, the outcome $E\left({Y}_{t}\right)$ refers to the observed daily PTB counts. $\propto$ is the intercept, ${Pollution}_{t}$ represents the pollutant concentration on day $t$, ${Temp}_{t}$ represents the temperature, ${RH}_{t}$ represents the relative humidity; ${Time}_{t}$ is time trend. $cb\left({Temp}_{t},lag\right)$, $cb\left({RH}_{t},lag\right)$ and $cb\left({Pollution}_{t},lag\right)$ indicates the matrix of temperature, relative humidity and air pollutants, respectively. Then, we use the DLNMs by the definition of a “cross-basis” function, a two dimensional function space expresing the influence of the predictor range and in its lag dimention. *ns()* denotes a natural cubic smooth spline function that removes unmeasured long-term and seasonal trend from the time series data set. ${DOW}_{t}$ represent the day of week; ${holiday}_{t}$ represent dummy variable (0 indicates non-holiday, and 1 indicates a holiday).

The maximum lag day was determined according to the Akaike information criterion for quasi-likelihood models (QAIC). The formula of QAIC was shown as follows:

$$QAIC=-\frac{2\;\ln\;\left(L\right)}c+2k$$

where $c$ is the variance expansion factor, $L$ is the likelihood function, and *k* is the formula parameters. In fact, the longer the lag time, the smaller QAIC would be, and too long a lag time might create a large bias. Therefore, we selected the maximum lag day up to 30 days according to the local minimum QAIC. Sensitivity analyses were made by changing the degree of freedom (df) for time (6–8 df/year). Finally, we selected the df of the natural cubic smooth splines of time were 7 per year in all the models. We assessmented relative risk (RR) and cumulative relative risk (CRR) for each ambient air pollutant in relation to preterm birth after an adjustment for two meteorological factors: daily mean value of temperature and relative humidity.

We used Microsoft Excel software and ArcGIS software 10.0 to organize and establish the dataset; the DLNM was employed by using the package dlnm Version 2.3.6 within R 4.0.1 software (Vienna, Australia).

## Results

### Baseline characteristics

Table 1 shows the daily descriptive results. During this time, the total number of births was 598,018, and there were 35,044 premature births. The daily new case of premature births was 16, ranging from 1 to a maximum of 48. Average concentration of PM_2.5_, PM_10_, SO_2_, NO_2_, O_3_, and CO in the six years was 42.44 μg/m^3^, 66.37 μg/m^3^, 10.21 μg/m^3^, 39.99 μg/m^3^, 39.75 μg/m^3^, and 916.08 μg/m^3^, respectively. The mean concentration of daily relative humidity and temperature was 75.12% and 19.92 °C, respectively.

**Table 1:** Summary of daily average concentrations of environmental variables and weather conditions in Chongqing

Variables	Mean ± SD	Min	Percentile			Max
			25th	50th	75th	
Preterm birth	16.00 ± 7.40	1.00	10.00	15.00	21.00	48.00
Air pollution						
PM_2.5_ (μg/m^3^)	42.44 ± 23.96	7.47	26.52	36.38	51.57	165.94
PM_10_ (μg/m^3^)	66.37 ± 32.03	13.06	44.80	59.85	79.91	228.76
SO_2_ (μg/m^3^)	10.21 ± 4.51	3.76	7.01	8.94	12.29	38.53
NO_2_ (μg/m^3^)	39.99 ± 11.35	12.24	31.76	38.26	47.18	81.82
O_3_ (μg/m^3^)	39.75 ± 25.25	4.41	20.41	33.53	53.52	142.65
CO (μg/m^3^)	916.08 ± 217.43	466.47	766.50	879.71	1025.88	2975.882
Meteorological factors						
Temperature (°C)	19.92 ± 7.81	1.20	12.70	20.30	26.30	36.50
Relative humidity (%)	75.12 ± 11.6	37.00	68.00	76.00	84.00	97.00

**Fig. 1:**
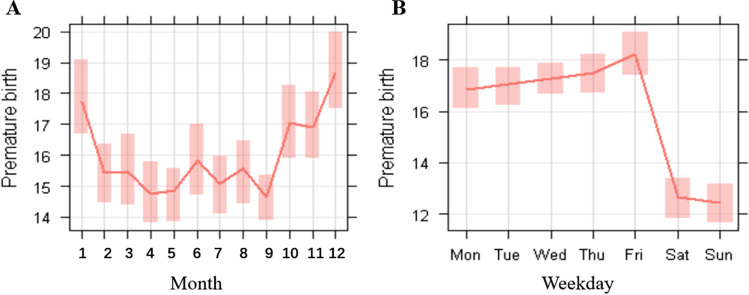
Distribution characteristics of monthly and weekly count of PTB in Chongqing, China, between 2015 and 2020. The two figures show the variation in the mean (**A** is the monthly average, and **B** is the weekly average), and the shading shows the extent to the 95% confidence interval for the mean

The number of PTB and mean concentration of air pollutants fluctuated with months and weeks. Except for O_3_, the average monthly concentration of air pollutants varied obviously with season, with low concentrations in summer and high concentrations in winter. What is more, the preterm birth also occurred more often in winter and less so in summer. To be specific, O_3_ peaked in the summer (Fig. 1, Supplementary Fig. 1).

### Spearman correlation

Table 2 depicts the correlation coefficient (*r*) between meteorological factors and air pollutants. The majority of the air pollutants (PM_2.5_, PM_10_, SO_2_, NO_2_, and CO) were positively and strongly correlated to others. The correlation between PM_2.5_ and PM_10_ was very close (*r* = 0.96). O_3_ was weekly and negatively correlated to other pollutants with r range from − 0.15 to − 0.51. Moreover, correlations were observed negative, moderate and significant between meteorological factors and air pollutants.

**Table 2:** Spearman coefficients between meteorological factors and daily ambient air pollutants in Chongqing, 2015–2020

	PM_2.5_	PM_10_	SO_2_	NO_2_	CO	O_3_	Temperature
PM_10_	0.96*						
SO_2_	0.64*	0.71*					
NO_2_	0.66*	0.73*	0.59*				
CO	0.71*	0.68*	0.59*	0.61*			
O_3_	− 0.30*	− 0.18*	− 0.15*	− 0.19*	− 0.47*		
Temperature	− 0.25*	− 0.17*	− 0.13*	− 0.21*	− 0.28*	0.51*	
Humidity	− 0.04	− 0.14*	− 0.21*	− 0.05*	0.12*	− 0.42*	− 0.46*

*P < 0.05.

### Associations between air pollutant exposure and PTB

**Fig. 2:**
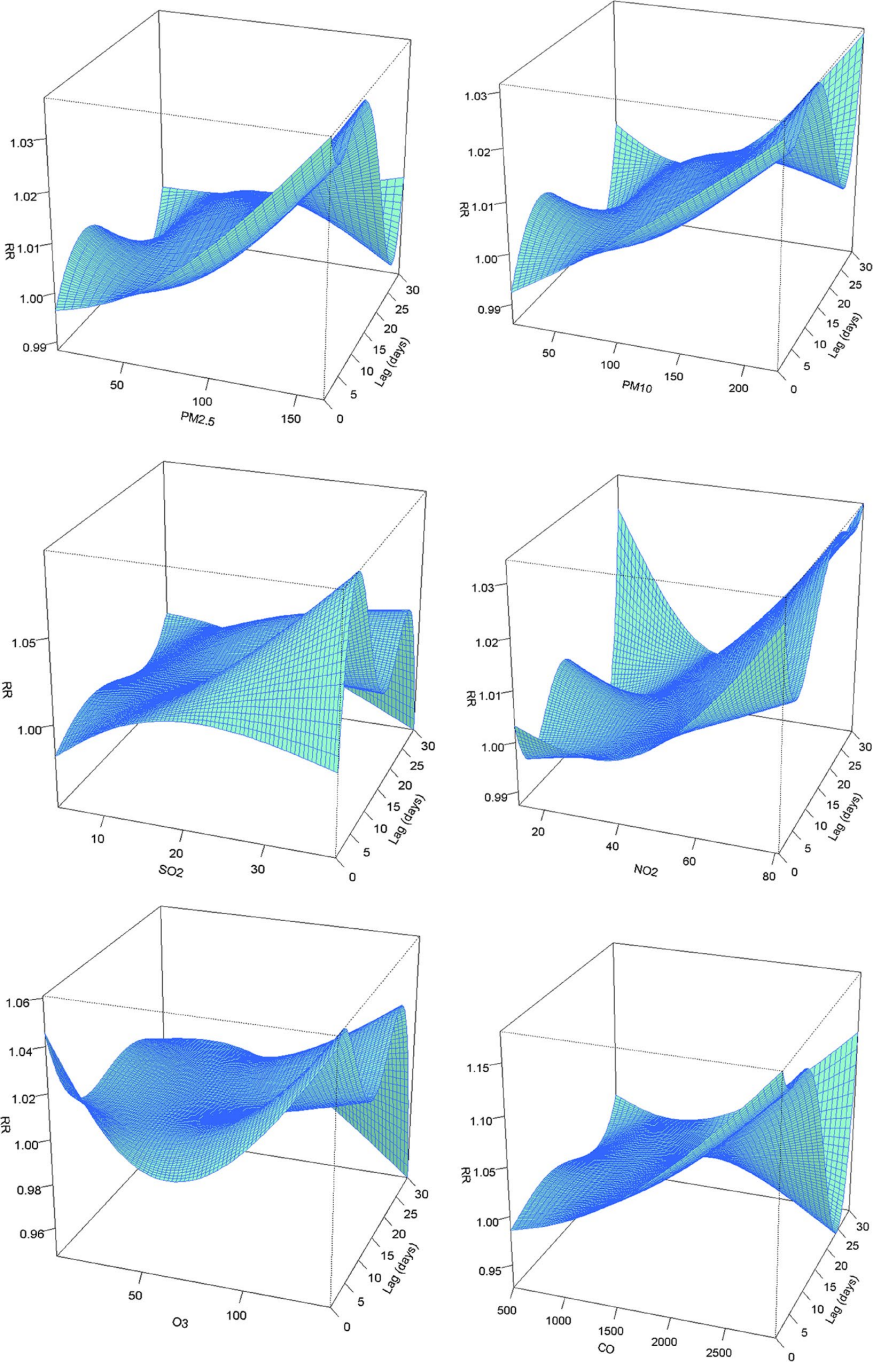
Three-dimensional (3D) lag-response curves specific to air pollutants for PTB

Figure 2 shows exposure-lag-response surfaces as RR describing the non-linear relationship between premature births and air pollutants along 30 lag days. The lag days is represented by one bottom edge of the cube, and the air pollution is represented by the other bottom edge. The height of the cube represents the RR of PTB.

**Fig. 3:**
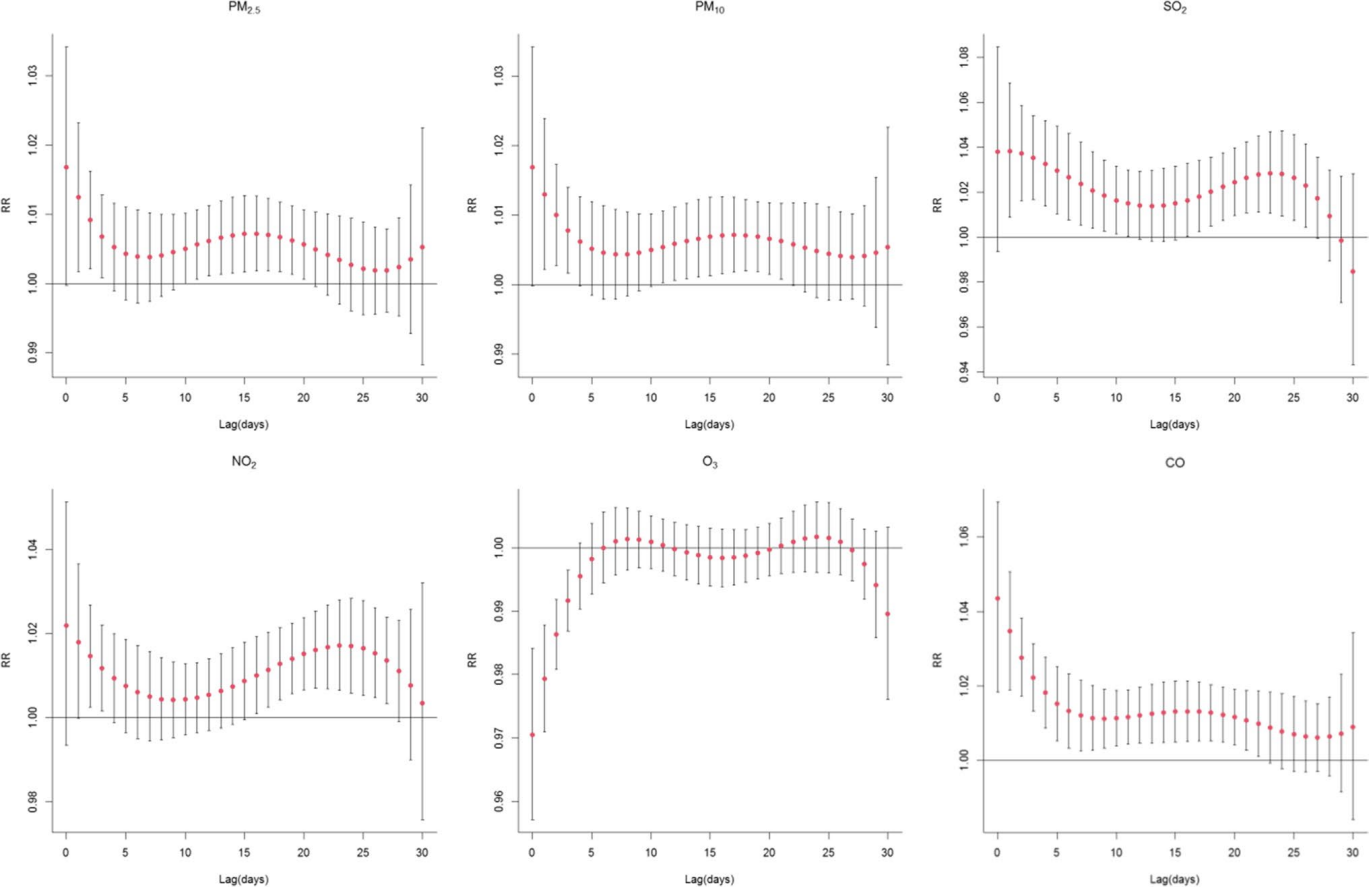
The lag-response relationship between PTB incidence and air pollutants at different lag days from 2015 to 2020. Reference value: PM_2.5_ at 42.44 μg/m^3^; PM_10_ at 66.37 μg/m^3^; SO_2_ at 10.21 μg/m^3^; NO_2_ at 39.99 μg/m^3^; O_3_ at 39.75 μg/m^3^; CO at 916.08 μg/m.^3^

Figure 3 demonstrates the RR and 95% CI of PTB with every 10 μg/m^3^ increase of each air pollutant in single-day (lag 0–30). The curve confirms a positive correlation between PM_2.5_ and PTB on lag 0–3 and lag 10–21 days, and the strongest influence in PTB associated with a 10 μg/m^3^ increase was at lag 0 (RR = 1.017, 95%CI: 1.000–1.034). The lag response curve of PM_10_ was similar to that of PM_2.5_, and the significant effect for PM_10_ on PTB was observed on lag 0–4 and lag 10–22 days. An obviously positive correlation between premature birth and maternal exposure with per 10 μg/m^3^ increase of SO_2_ was observed on lag 1–11 and lag 16–17 days. For NO_2_, an increased risk of PTB was observed on lag 0–3 and lag 16–27 days and peaking at the lag day 23 (RR = 1.017, 95%CI: 1.007–1.027).

O_3_ had a negative correlation with the risk of PTB between 0 and 4 days. Short-term exposure to CO did increase the risk of PTB, and the maximum RR values of CO with a 100 μg/m^3^ increment was found at lag 0 (RR = 1.044, 95%CI: 1.018–1.069) and then fell gradually. By lag day 23, the effect of CO was no longer present (see Table 1 of Supplementary Information).

**Fig. 4:**
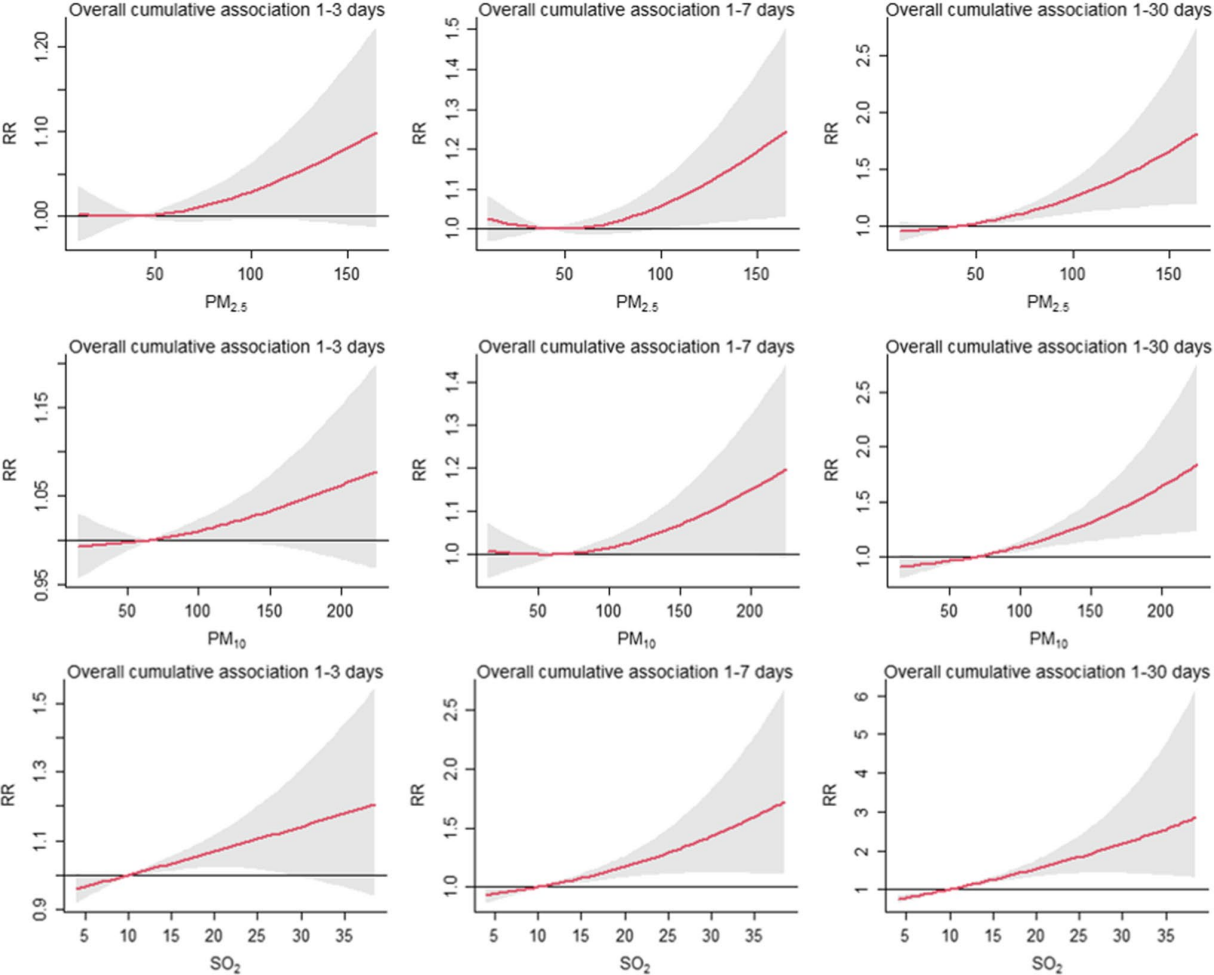

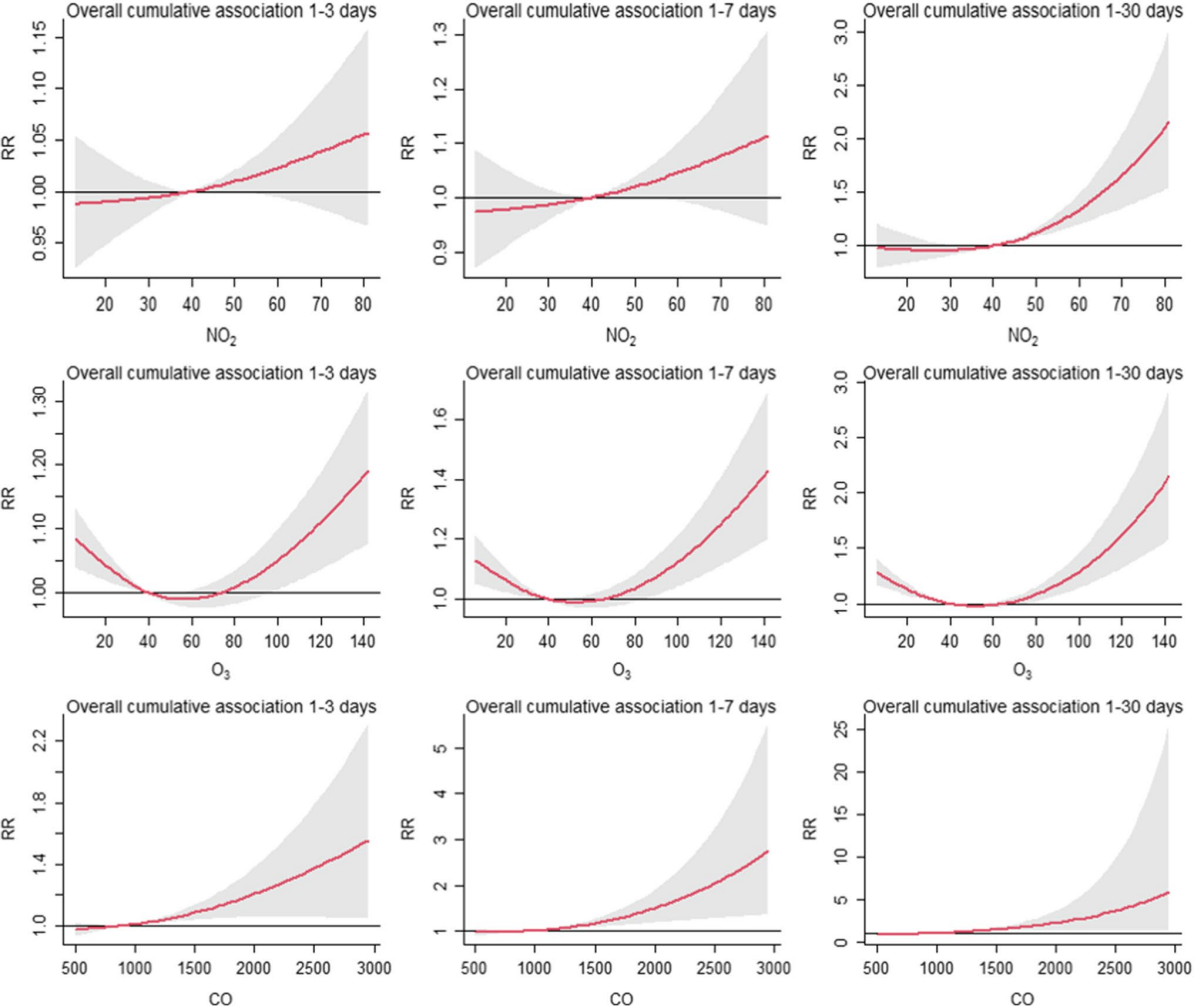
Exposure–response curves for the effect of air pollutants on daily counts of PTB at 1–3, 1–7, and 1–30 lag days. *X*-axis: the concentration of each air pollutant; *y*-axis: the relative risk of air pollution on PTB. Reference value: PM_2.5_ at 42.44 μg/m^3^; PM_10_ at 66.37 μg/m^3^; SO_2_ at 10.21 μg/m^3^; NO_2_ at 39.99 μg/m^3^; O_3_ at 39.75 μg/m^3^; CO at 916.08 μg/m.^3^

Figure 4 shows the overall CRR of air pollutants exposure and PTB with a lag of 1–3 days, 1–7 days, and 1–30 days. Analysis of the relationship revealed that the overall cumulative relative risk (CRR) of PTB had an approximately J-shape with PM_2.5_ and PM_10_. The cumulative relative risk of PM_2.5_ over 100 μg/m^3^ (CRR = 1.058, 95% CI: 1.000–1.120) from lag 1–7 and over 50 μg/m^3^ (CRR = 1.021, 95%CI: 1.006–1.036) from 1 to 30 was statistically significant. Similarly, the cumulative relative risk of PM_10_ over 70 μg/m^3^ (CRR = 1.010, 95%CI: 1.003–1.016) from lag 1–30 was significant. The highest risk of SO_2_ from lag 1–3 days was at 20 μg/m^3^ (CRR = 1.069, 95% CI: 1.023–1.117) during 15–25 μg/m^3^. The cumulative relative risk of SO_2_ over 15 μg/m^3^ from lag 1–7 (CRR = 1.074, 95% CI: 1.035–1.115) and lag 1–30 (CRR = 1.252, 95% CI: 1.174–1.335) were statistically significant. The 1–30 days cumulative relative risk effect of NO_2_ was statistically significant at a concentration above 45 μg/m^3^. Notably, the overall cumulative relative risk of O_3_ was a U-shape. When the concentration was lower than 30 μg/m^3^ or higher than 80 μg/m^3^, the CRR value was statistically significant, and the concentration between 40 and 70 μg/m^3^ negatively correlated with PTB. The cumulative relative risk effect of CO exposure showed that CRR increased significantly after 1000 μg/m^3^, with significant statistical significance in lag for 1–3 days, 1–7 days, and 1–30 days (Table 3).

**Table 3:** The overall cumulative association between air pollutants and PTB

Level (μg/m^3^)	Lag 1–3 days		Lag 1–7 days		Lag 1–30 days	
	RR	95%CI	RR	95%CI	RR	95%CI
PM_2.5_						
20	1.001	(0.980, 1.022)	1.014	(0.978, 1.051)	0.960	(0.904, 1.020)
40	1.000	(0.998, 1.001)	1.001	(0.998, 1.003)	0.995	(0.991, 1.000)
60	1.004	(0.994, 1.015)	1.004	(0.986, 1.022)	**1.053**	**(1.022, 1.086)**
80	1.014	(0.994, 1.035)	1.024	(0.989, 1.059)	**1.138**	**(1.066, 1.214)**
100	1.029	(0.996, 1.063)	**1.058**	**(1.000, 1.120)**	**1.250**	**(1.110, 1.408)**
120	1.048	(0.996, 1.103)	**1.105**	**(1.011, 1.209)**	**1.391**	**(1.144, 1.692)**
140	1.069	(0.992, 1.152)	**1.163**	**(1.020, 1.326)**	**1.562**	**(1.170, 2.084)**
160	1.093	(0.988, 1.209)	**1.229**	**(1.028, 1.468)**	**1.763**	**(1.192, 2.606)**
PM_10_						
20	0.993	(0.961, 1.026)	1.005	(0.950, 1.064)	0.919	(0.827, 1.021)
40	0.995	(0.979, 1.012)	1.000	(0.972, 1.029)	0.949	(0.901, 1.001)
60	0.999	(0.996, 1.002)	0.999	(0.994, 1.005)	0.987	(0.977, 0.997)
80	1.004	(0.997, 1.010)	1.004	(0.994, 1.015)	**1.036**	**(1.016, 1.056)**
100	1.010	(0.997, 1.023)	1.016	(0.993, 1.039)	**1.099**	**(1.052, 1.147)**
120	1.018	(0.997, 1.039)	1.033	(0.997, 1.071)	**1.176**	**(1.093, 1.265)**
140	1.028	(0.996, 1.060)	**1.056**	**(1.001, 1.115)**	**1.268**	**(1.131, 1.422)**
160	1.038	(0.992, 1.086)	**1.084**	**(1.002, 1.172)**	**1.377**	**(1.163, 1.630)**
180	1.049	(0.986, 1.117)	**1.115**	**(1.000, 1.243)**	**1.502**	**(1.189, 1.896)**
200	1.062	(0.979, 1.151)	1.150	(0.998, 1.326)	**1.644**	**(1.211, 2.231)**
220	1.074	(0.970, 1.189)	1.188	(0.993, 1.420)	**1.803**	**(1.230, 2.642)**
SO_2_						
5	0.966	(0.933, 1.001)	0.944	(0.886, 1.005)	0.780	(0.699, 0.869)
10	1.000	(1.000, 1.000)	1.000	(1.000, 1.000)	1.000	(1.000, 1.000)
15	**1.034**	**(1.013, 1.056)**	**1.074**	**(1.035, 1.115)**	**1.252**	**(1.174, 1.335)**
20	**1.069**	**(1.023, 1.117)**	**1.170**	**(1.082, 1.266)**	**1.531**	**(1.335, 1.756)**
25	**1.105**	**(1.017, 1.200)**	**1.289**	**(1.112, 1.494)**	**1.838**	**(1.418, 2.382)**
30	1.141	(0.996, 1.308)	**1.430**	**(1.120, 1.825)**	**2.000**	**(1.419, 3.336)**
35	1.179	(0.966, 1.438)	**1.595**	**(1.116, 2.281)**	**2.554**	**(1.367, 4.773)**
NO_2_						
15	0.989	(0.932, 1.048)	0.975	(0.882, 1.078)	0.975	(0.807, 1.177)
20	0.990	(0.948, 1.034)	0.978	(0.909, 1.053)	0.962	(0.838, 1.105)
25	0.992	(0.963, 1.021)	0.982	(0.935, 1.032)	0.955	(0.871, 1.047)
30	0.994	(0.977, 1.010)	0.987	(0.959, 1.015)	0.956	(0.908, 1.008)
35	0.996	(0.989, 1.003)	0.993	(0.981, 1.004)	0.970	(0.949, 0.991)
40	1.000	(1.000, 1.000)	1.000	(1.000, 1.000)	1.000	(1.000, 1.000)
45	1.005	(0.999, 1.010)	**1.009**	**(1.000, 1.018)**	**1.050**	**(1.033, 1.068)**
50	1.010	(0.999, 1.021)	**1.020**	**(1.001, 1.039)**	**1.122**	**(1.081, 1.165)**
55	1.016	(0.998, 1.035)	**1.032**	**(1.000, 1.066)**	**1.218**	**(1.139, 1.301)**
60	1.023	(0.995, 1.053)	1.046	(0.995, 1.100)	**1.338**	**(1.204, 1.486)**
65	1.031	(0.990, 1.074)	1.061	(0.987, 1.141)	**1.485**	**(1.276, 1.728)**
70	1.039	(0.983, 1.097)	1.077	(0.976, 1.188)	**1.661**	**(1.354, 2.039)**
75	1.047	(0.976, 1.124)	1.094	(0.964, 1.241)	**1.869**	**(1.437, 2.430)**
80	1.056	(0.968, 1.152)	1.111	(0.951, 1.298)	**2.110**	**(1.527, 2.914)**
O_3_						
10	**1.072**	**(1.034, 1.112)**	**1.110**	**(1.044, 1.180)**	**1.242**	**(1.147, 1.346)**
20	**1.043**	**(1.019, 1.067)**	**1.063**	**(1.024, 1.104)**	**1.136**	**(1.083, 1.191)**
30	**1.018**	**(1.008, 1.028)**	**1.025**	**(1.008, 1.043)**	**1.053**	**(1.032, 1.075)**
40	1.000	(1.000, 1.000)	1.000	(1.000, 1.000)	1.000	(1.000, 1.000)
50	0.991	(0.983, 0.998)	0.990	(0.977, 1.002)	0.979	(0.966, 0.993)
60	0.990	(0.976, 1.003)	0.993	(0.970, 1.016)	0.988	(0.963, 1.013)
70	0.996	(0.977, 1.015)	1.009	(0.976, 1.043)	1.023	(0.983, 1.064)
80	1.008	(0.982, 1.035)	1.036	(0.991, 1.084)	**1.084**	**(1.021, 1.152)**
90	1.027	(0.992, 1.062)	**1.075**	**(1.014, 1.140)**	**1.173**	**(1.074, 1.281)**
100	**1.050**	**(1.005, 1.097)**	**1.124**	**(1.042, 1.212)**	**1.290**	**(1.142, 1.458)**
110	**1.078**	**(1.020, 1.140)**	**1.182**	**(1.074, 1.301)**	**1.439**	**(1.224, 1.690)**
120	**1.110**	**(1.036, 1.189)**	**1.250**	**(1.110, 1.407)**	**1.621**	**(1.321, 1.988)**
130	**1.145**	**(1.054, 1.244)**	**1.326**	**(1.150, 1.529)**	**1.840**	**(1.433, 2.362)**
140	**1.183**	**(1.073, 1.304)**	**1.410**	**(1.192, 1.667)**	**2.096**	**(1.557, 2.822)**
CO						
500	0.974	(0.933, 1.018)	0.972	(0.900, 1.049)	0.833	(0.729, 0.952)
1000	**1.008**	**(1.003, 1.014)**	**1.014**	**(1.005, 1.023)**	**1.046**	**(1.031, 1.062)**
1500	**1.085**	**(1.037, 1.134)**	**1.178**	**(1.092, 1.272)**	**1.464**	**(1.250, 1.715)**
2000	**1.206**	**(1.055, 1.379)**	**1.503**	**(1.190, 1.899)**	**2.249**	**(1.366, 3.704)**
2500	**1.372**	**(1.053, 1.786)**	**2.038**	**(1.284, 3.234)**	**3.676**	**(1.377, 9.812)**
2900	**1.533**	**(1.045, 2.248)**	**2.661**	**(1.360, 5.206)**	**5.573**	**(1.344, 23.104)**

We estimated the effect of each air pollutant per 1 μg/m^3^ on PTB. The concentration of air pollutants varies widely, so we chose to show concentration to make the table more concise. More detail results are available in Supplementary Table 2. Boldface indicated statistical significance established at *P* < 0.05

## Discussion

This study suggested that the daily exposures to PM_2.5_, PM_10_, SO_2_, CO, and NO_2_ and during pregnancy were positively correlated with the increased risk of PTB after adjusting for mean relative humidity and temperature at lag 0–30 days. At the time of this study, both the levels of PM_2.5_ and PM_10_ were exceeded the first-level of National Air Quality Standards (NAQS) values (PM_2.5_: 35 μg/m^3^; PM_10_: 50 μg/m^3^). That means the particulate matter pollution was quite severe in Chongqing, China. Furthermore, compared with developed countries, China had a longer particulate matter exposure duration and a higher magnitude (Guan et al. 2016; Sun et al. 2021). The levels of CO, O_3_, NO_2_, and SO_2_ were below the primary standard (CO: 4000 μg/m^3^; O_3_: 100 μg/m^3^; NO_2_: 80 μg/m^3^; SO_2_: 50 μg/m^3^) in the NAQS. As far as we know, this is the first study to assess the impact of short-term air pollutants exposure on PTB with such a pollution level in Chongqing.

Previous researches suggested that the risk of preterm births (PTBs) following prenatal exposure to air pollution was inconclusive. Huang et al. found that an interquartile range increases in NO_2_, SO_2_ and O_3_ were related with 0.46% (95%CI: − 0.25 ~ 1.23), 0.37% (95%CI: − 1.77 ~ 2.57) and 2.09% (95%CI: − 8.00 ~ 13.29) increase risk for PTB at lag 2, respectively (Huang et al. 2020). One large study in Changsha, China, covering 78 midwifery institutions and 344,880 live births, reported that NO_2_ was associated with PTBs on lag 0–2, lag 4, and lag 5 (Xiong et al. 2019). In another study, Lee et al. suggested that cumulative exposure to O_3_ and PM_10_ from 0 to 6 days before birth was not associated with the risk of PTBs (Lee et al. 2008). The threshold for the impact of air pollution is generally expected to protect people’s health by controlling pollutants below this concentration (Li et al. 2016). Fleischer et al. reported that the possible threshold effect of PM_2.5_ on PTB is 36.5 mg/m^3^ (Fleischer et al. 2014). DeFranco et al. observed that maternal exposure to high concentrations of PM_2.5_ in excess of 15 μg/m^3^ was associated with PTB significantly (DeFranco et al. 2016). A Spanish study observed that perinatal exposure to a certain high concentration of traffic-related air pollution (such as NO_2_ > 46.2 mg/m^3^) was associated with PTB (Llop et al. 2010). While, the results of a Beijing research suggested that there was a correlation between pollutants and preterm birth, but with no evidence of a threshold (Guan et al. 2019).

In this paper, short-term air pollution exposure was significantly associated with a higher risk of PTB in few days before birth. For a 10 μg/m^3^ increment in PM_2.5_ concentration, the strongest effect on PTB was on lag 0 day (RR = 1.017, 95%CI: 1.000–1.014), which was higher than in Xuzhou and Beijing (Guan et al. 2019; Li et al. 2021b). Similarly, exposure to PM_10_ can also acutely affect PTB. Moreover, Leem et al. observed that exposure to SO_2_ in the last trimester of maternal with percutaneous transluminal dilatation (PTD) was statistically significant (Leem et al. 2006). Moreover, our results showed that exposure to SO_2_ positively correlated with premature birth, and SO_2_ has a long effect of 20 days. There was a positive correlation between PTB and NO_2_ on lag 0–3 and 16–27 days, and peaking on the 23th day. In contrast, Ji et al. suggested that the relationship between PTB and NO_2_ exposure was not significant during the first and second trimesters, compared to significant correlations in the last week and last month before labor (Ji et al. 2019). We found the highest risk for PTB occurred with CO in the short term, and the relative risk value was highest at lag 0 (RR = 1.119, 95%CI: 1.049–1.194). The proposed mechanism of action related to tissue oxygenation, especially CO binding to fetal hemoglobin to reduce the availability of oxygen. The unexpected negative relationship between O_3_ and PTB was found between the 0–4 lag days. This may be interpreted by the inverse association between O_3_ and other assessed air pollutants (Nobles et al. 2019; Reynolds et al. 2019). Conversely, some studies found that O_3_ and CO were not associated with PTB (Guan et al. 2019; Liu et al. 2019). Interestingly, we observed that although the relative risk of PM_2.5_, PM_10_, NO_2_, SO_2_, and CO fluctuated with lag days, and the overall relative risk did show a downward trend.

This current study indicates that the cumulative relative risk of air pollutants increases with the increase of lag days, with evidence of a threshold. The cumulative relative risk of PM_2.5_ and PM_10_ exposure lags 1–7 days and 1–30 days, showing that the effect was strong after 50 μg/m^3^. In Huang’s analysis, when air pollution level was high in terms of PM_2.5_ (75 μg/m^3^), the risk of PTB was higher, and the curve presented as a rapid growth (Huang et al. 2020). The cumulative relative risk of SO_2_ over 15 μg/m^3^ from lag 1–7 and lag 1–30 was statistically significant. Moreover, in Li’s study, the curve of SO_2_ was similar to that in our analysis, but the relationships between PM_10_ and PM_2.5_ were not significant (Li et al. 2016). Increased concentrations of NO_2_ above 45 μg/m^3^ were positively associated with an increased risk of PTB in this paper. However, Llop’s study suggested that exposure to NO_2_ above 46.2 mg/m^3^ was associated with PTB (Llop et al. 2010). Our analysis found that the associations between O_3_ and PTB were generally U-shaped. That is, a threshold effect of O_3_ was indicated. Exposure to CO has the strongest effect on the occurrence of PTB, and the cumulative relative effect increases greatly after CO level over 1000 μg/m^3^, whether there is a lag of 1–3 days or 1–7 days or 1–30 days. In addition, we found the overall accumulative relative curves relatively flattened out at low levels and were steep at higher levels, which is inconsistent with the results in previous researches (Giorgis-Allemand et al. 2017; Guan et al. 2019; Pope et al. 2009). However, it is worth mentioning that we should be cautious about this result because high levels of air pollution are rarely observed.

Associations between air pollutants short-term exposures and risk for PTBs may indicate that air pollutants could trigger the biologic mechanism of parturition quickly, bringing out PTB in the following days. There are several potential biologic mechanisms that could support this association via a series of causes, such as inflammation, endocrine disruption, oxidative stress, blood coagulation, and hemodynamic responses (Kumar et al. 2019; Li et al. 2008; Pope et al. 2004; Slama et al. 2008). Air pollutants can translocate to the placenta through villous tissue and thus lead to preterm premature rupture of membranes (PPROM) (Bove et al. 2019; Li et al. 2021a). Simultaneously, when air pollutants are inhaled, cytokines trigger oxidative stress, which can cause endothelial dysfunction and the development of pregnancy preeclampsia (Yorifuji et al. 2015). In addition, trace metals and polycyclic aromatic hydrocarbons (PAHs) bound to particulate matter may create potential health risk (Ambade et al. 2022a, 2022b; Kumar et al. 2020; Vithanage et al. 2022).

There are many underlying factors responsible for the different results found from study to study. Firstly, air pollution levels in Chongqing were higher than in most literature. From 2015 to 2020, the concentrations of PM_2.5_ and PM_10_ were 42.44 μg/m^3^ and 66.37 μg/m^3^ in Chongqing, which exceeded the NAQS standard. Secondly, the social and demographic conditions of the inhabitants of each area may be diverse, such as lifestyles, disease patterns, or genetic backgrounds. Thirdly, the study period, air pollutant unit and statistical model were selected differently in the other literature.

Compared to the previous studies, when analyzing the relationship between premature birth and air pollution, our study has several advantages. We used a large sample size of nearly 600,000 total births and 35,044 premature delivery cases. This number is at least an order of magnitude more than offered in the previous research papers on this topic (Chen et al. 2021; Ji et al. 2019; Li et al. 2021b, 2019). The dataset included the total number of all eligible birth actual occurrences in study areas, covering air pollution data gathered from 17 air monitoring sites in Chongqing’s main urban area, which effectively reduced any selection bias. Moreover, the application of weekly or trimestral data had to face the fact that the effect of extreme pollution events would be underestimated, thereby averaging their effects over long time scales. Additionally, the spatial variability of exposure can be effectively reflected by the individual exposure estimation based on the detailed home address of the mother in this study.

There were some limitations to mention. First of all, we obtained the air pollution data from available monitors that may not fully represent the maternal exposure level. The measured value may overestimate the maternal exposure level because pregnant women spend most of their time indoors. Secondly, the individual risk factors, such as maternal health status and maternal age, were not adjusted because of information shortage. Future studies should include personal risk factors because of their potential changes for the correlation between PTB and air pollution (Kingsley et al. 2017). Moreover, we did not analyze the relationship between air pollutants and very preterm birth (VPTB) in this study. Some researches demonstrated that air pollution can also increase the incidence of VPTB (Guo et al. 2018; Ju et al. 2021; Wang et al. 2018). Meanwhile, we only studied the short-term effects of a single pollutant and PTBs, and the interactive effect of air pollutants also deserves further attention. Therefore, we cannot conclude whether the interactive effect of air pollutants introduced potential bias or just simply confounded effects with each other.

## Conclusion

In conclusion, we found that maternal air pollution exposure had short-term and delayed effects on PTB, thereby increasing the risk of PTB. This study provides evidence from a study of a large population that reducing air pollution level to a certain threshold might greatly benefit birth outcomes. Pregnant women should be aware of the risk of air pollution and avoid exposure to high levels, if possible, especially in the last few weeks of pregnancy. This study thus has particular important public health significance for policy makers who design and implement air pollution preventive measures.

## Supplementary Information

Below is the link to the electronic supplementary material.

Supplementary file1 (DOCX 444 KB)

## Data Availability

The datasets that support the findings of this study are openly available from the corresponding author on reasonable request.

## Abbreviations

PTB: Preterm birth
PM_2.5_: Particulate matter 2.5
PM_10_: Particulate 10
SO_2_: Sulfur dioxide
NO_2_: Nitrogen dioxide
CO: Carbon monoxide
O_3_: Ozone
DLNM: Distributed lag non-linear models
SD: Standard deviation
IQR: Inter-quartile range
RR: Relative risk
CRR: Cumulative relative risk
CI: Confidence interval
