# Supplementary material for: The short-term effects of air pollution exposure on preterm births in Chongqing, China: 2015–2020
Source: Environ Sci Pollut Res Int. 2023 Feb 22;30(18):51679–91. doi: 10.1007/s11356-023-25624-2 (PMC10119072; doi:10.1007/s11356-023-25624-2)
Supplement: Supplementary file 1 — Supplementary file1 (DOCX 444 KB) [file 11356_2023_25624_MOESM1_ESM.docx]

Supplementary table 1-1. The relative risk of preterm birth and air pollution (PM_2.5_，PM_10_, and SO_2_) from distribute lag models

| Lag, d |  | PM_2.5_ | |  | PM_10_ | |  | SO_2_ | |
| --- | --- | --- | --- | --- | --- | --- | --- | --- | --- |
|  |  | RR | 95%CI |  | RR | 95%CI |  | RR | 95%CI |
| 0 |  | 1.017 | （1.000，1.034） | | 1.017 | （1.000，1.034） | | 1.038 | （0.994，1.085） |
| 1 |  | 1.012 | （1.002，1.023） | | 1.013 | （1.002，1.024） | | 1.038 | （1.009，1.069） |
| 2 |  | 1.009 | （1.002，1.016） | | 1.010 | （1.003，1.017） | | 1.037 | （1.016，1.059） |
| 3 |  | 1.007 | （1.001，1.013） | | 1.008 | （1.002，1.014） | | 1.035 | （1.017，1.054） |
| 4 |  | 1.005 | （0.999，1.012） | | 1.006 | （1.000，1.013） | | 1.033 | （1.014，1.052） |
| 5 |  | 1.004 | （0.998，1.011） | | 1.005 | （0.999，1.012） | | 1.030 | （1.010，1.049） |
| 6 |  | 1.004 | （0.997，1.011） | | 1.005 | （0.998，1.011） | | 1.027 | （1.008，1.046） |
| 7 |  | 1.004 | （0.997，1.010） | | 1.004 | （0.998，1.011） | | 1.024 | （1.005，1.042） |
| 8 |  | 1.004 | （0.998，1.010） | | 1.004 | （0.998，1.010） | | 1.021 | （1.004，1.038） |
| 9 |  | 1.005 | （0.999，1.010） | | 1.005 | （0.999，1.010） | | 1.018 | （1.003，1.034） |
| 10 |  | 1.005 | （1.000，1.010） | | 1.005 | （1.000，1.010） | | 1.016 | （1.001，1.032） |
| 11 |  | 1.006 | （1.001，1.011） | | 1.005 | （1.000，1.011） | | 1.015 | （1.000，1.030） |
| 12 |  | 1.006 | （1.001，1.011） | | 1.006 | （1.001，1.011） | | 1.014 | （0.999，1.029） |
| 13 |  | 1.007 | （1.001，1.012） | | 1.006 | （1.001，1.012） | | 1.014 | （0.998，1.030） |
| 14 |  | 1.007 | （1.002，1.012） | | 1.007 | （1.001，1.012） | | 1.014 | （0.998，1.031） |
| 15 |  | 1.007 | （1.002，1.013） | | 1.007 | （1.001，1.013） | | 1.015 | （0.999，1.032） |
| 16 |  | 1.007 | （1.002，1.013） | | 1.007 | （1.002，1.013） | | 1.016 | （1.000，1.033） |
| 17 |  | 1.007 | （1.002，1.012） | | 1.007 | （1.002，1.013） | | 1.018 | （1.002，1.034） |
| 18 |  | 1.007 | （1.002，1.012） | | 1.007 | （1.002，1.012） | | 1.020 | （1.005，1.036） |
| 19 |  | 1.006 | （1.001，1.011） | | 1.007 | （1.002，1.012） | | 1.022 | （1.008，1.038） |
| 20 |  | 1.006 | （1.001，1.011） | | 1.007 | （1.002，1.012） | | 1.025 | （1.010，1.040） |
| 21 |  | 1.005 | （1.000，1.010） | | 1.006 | （1.001，1.012） | | 1.026 | （1.011，1.042） |
| 22 |  | 1.004 | （0.998，1.010） | | 1.006 | （1.000，1.012） | | 1.028 | （1.011，1.045） |
| 23 |  | 1.003 | （0.997，1.010） | | 1.005 | （0.999，1.012） | | 1.029 | （1.011，1.047） |
| 24 |  | 1.003 | （0.996，1.009） | | 1.005 | （0.998，1.012） | | 1.028 | （1.009，1.047） |
| 25 |  | 1.002 | （0.996，1.009） | | 1.004 | （0.998，1.011） | | 1.026 | （1.007，1.046） |
| 26 |  | 1.002 | （0.996，1.008） | | 1.004 | （0.998，1.011） | | 1.023 | （1.005，1.042） |
| 27 |  | 1.002 | （0.996，1.008） | | 1.004 | （0.998，1.010） | | 1.017 | （1.000，1.036） |
| 28 |  | 1.002 | （0.995，1.009） | | 1.004 | （0.997，1.011） | | 1.009 | （0.989，1.030） |
| 29 |  | 1.003 | （0.993，1.014） | | 1.005 | （0.994，1.015） | | 0.999 | （0.971，1.027） |
| 30 |  | 1.005 | （0.988，1.023） | | 1.005 | （0.988，1.023） | | 0.985 | （0.943，1.028） |

Supplementary table 1-2. The relative risk of preterm birth and air pollution (NO_2_，O_3_, and CO) from distribute lag models

| Lag, d |  | NO_2_ | |  | O_3_ | |  | CO | |
| --- | --- | --- | --- | --- | --- | --- | --- | --- | --- |
|  |  | RR | 95%CI |  | RR | 95%CI |  | RR | 95%CI |
| 0 |  | 1.022 | （0.993，1.051） | | 0.971 | （0.950，0.991） | | 1.044 | （1.018，1.069） |
| 1 |  | 1.018 | （1.000，1.036） | | 0.979 | （0.967，0.992） | | 1.035 | （1.019，1.051） |
| 2 |  | 1.015 | （1.003，1.027） | | 0.986 | （0.978，0.995） | | 1.028 | （1.017，1.038） |
| 3 |  | 1.012 | （1.002，1.022） | | 0.992 | （0.984，0.999） | | 1.022 | （1.013，1.031） |
| 4 |  | 1.009 | （0.999，1.020） | | 0.996 | （0.988，1.004） | | 1.018 | （1.009，1.028） |
| 5 |  | 1.007 | （0.996，1.019） | | 0.998 | （0.990，1.007） | | 1.015 | （1.005，1.025） |
| 6 |  | 1.006 | （0.995，1.017） | | 1.000 | （0.992，1.009） | | 1.013 | （1.003，1.023） |
| 7 |  | 1.005 | （0.994，1.016） | | 1.001 | （0.993，1.009） | | 1.012 | （1.003，1.022） |
| 8 |  | 1.004 | （0.995，1.014） | | 1.001 | （0.994，1.009） | | 1.011 | （1.003，1.020） |
| 9 |  | 1.004 | （0.995，1.013） | | 1.001 | （0.995，1.008） | | 1.011 | （1.003，1.019） |
| 10 |  | 1.004 | （0.996，1.013） | | 1.001 | （0.995，1.007） | | 1.011 | （1.004，1.019） |
| 11 |  | 1.005 | （0.996，1.013） | | 1.000 | （0.994，1.007） | | 1.012 | （1.004，1.019） |
| 12 |  | 1.005 | （0.997，1.014） | | 1.000 | （0.993，1.006） | | 1.012 | （1.005，1.020） |
| 13 |  | 1.006 | （0.998，1.015） | | 0.999 | （0.993，1.006） | | 1.013 | （1.005，1.020） |
| 14 |  | 1.007 | （0.998，1.017） | | 0.999 | （0.992，1.006） | | 1.013 | （1.005，1.021） |
| 15 |  | 1.009 | （0.999，1.018） | | 0.999 | （0.992，1.006） | | 1.013 | （1.005，1.021） |
| 16 |  | 1.010 | （1.001，1.019） | | 0.998 | （0.991，1.005） | | 1.013 | （1.005，1.021） |
| 17 |  | 1.011 | （1.003，1.020） | | 0.998 | （0.992，1.005） | | 1.013 | （1.005，1.021） |
| 18 |  | 1.013 | （1.004，1.021） | | 0.999 | （0.992，1.005） | | 1.013 | （1.005，1.020） |
| 19 |  | 1.014 | （1.006，1.022） | | 0.999 | （0.993，1.005） | | 1.012 | （1.005，1.020） |
| 20 |  | 1.015 | （1.007，1.024） | | 1.000 | （0.993，1.006） | | 1.012 | （1.004，1.019） |
| 21 |  | 1.016 | （1.007，1.025） | | 1.000 | （0.994，1.007） | | 1.011 | （1.003，1.019） |
| 22 |  | 1.017 | （1.007，1.027） | | 1.001 | （0.994，1.008） | | 1.010 | （1.001，1.019） |
| 23 |  | 1.017 | （1.006，1.028） | | 1.001 | （0.993，1.010） | | 1.009 | （0.999，1.018） |
| 24 |  | 1.017 | （1.006，1.028） | | 1.002 | （0.993，1.010） | | 1.008 | （0.998，1.018） |
| 25 |  | 1.016 | （1.005，1.028） | | 1.002 | （0.993，1.010） | | 1.007 | （0.997，1.017） |
| 26 |  | 1.015 | （1.005，1.026） | | 1.001 | （0.993，1.009） | | 1.006 | （0.997，1.016） |
| 27 |  | 1.014 | （1.003，1.024） | | 1.000 | （0.992，1.007） | | 1.006 | （0.997，1.015） |
| 28 |  | 1.011 | （0.999，1.023） | | 0.997 | （0.989，1.006） | | 1.006 | （0.996，1.017） |
| 29 |  | 1.008 | （0.990，1.026） | | 0.994 | （0.981，1.007） | | 1.007 | （0.992，1.023） |
| 30 |  | 1.003 | （0.976，1.032） | | 0.990 | （0.969，1.011） | | 1.009 | （0.984，1.034） |

Supplementary table 2-1. The overall cumulative association between preterm birth and PM_2.5_

| Level (μg/m^3^) |  | PM_2.5_ & 1-3 | |  | PM_2.5_ & 1-7 | |  | PM_2.5_ & 1-30 | |
| --- | --- | --- | --- | --- | --- | --- | --- | --- | --- |
|  |  | RR | 95%CI |  | RR | 95%CI |  | RR | 95%CI |
| 10 |  | 1.002 | （0.970，1.036） |  | 1.024 | （0.969，1.083） | | 0.947 | （0.862，1.041） |
| 20 |  | 1.001 | （0.980，1.022） |  | 1.014 | （0.978，1.051） | | 0.960 | （0.904，1.020） |
| 30 |  | 1.000 | （0.989，1.010） |  | 1.006 | （0.988，1.024） | | 0.976 | （0.947，1.005） |
| 40 |  | 1.000 | （0.998，1.001） |  | 1.001 | （0.998，1.003） | | 0.995 | （0.991，1.000） |
| 50 |  | 1.001 | （0.996，1.007） |  | 1.000 | （0.991，1.009） | | 1.021 | （1.006，1.036） |
| 60 |  | 1.004 | （0.994，1.015） |  | 1.004 | （0.986，1.022） | | 1.053 | （1.022，1.086） |
| 70 |  | 1.009 | （0.993，1.024） |  | 1.012 | （0.986，1.038） | | 1.092 | （1.043，1.143） |
| 80 |  | 1.014 | （0.994，1.035） |  | 1.024 | （0.989，1.059） | | 1.138 | （1.066，1.214） |
| 90 |  | 1.021 | （0.996，1.048） |  | 1.039 | （0.995，1.086） | | 1.190 | （1.089，1.301） |
| 100 |  | 1.029 | （0.996，1.063） |  | 1.058 | （1.000, 1.120） |  | 1.250 | （1.110，1.408） |
| 110 |  | 1.038 | （0.996，1.082） |  | 1.080 | （1.006，1.160） |  | 1.317 | （1.128，1.537） |
| 120 |  | 1.048 | （0.996，1.103） |  | 1.105 | （1.011，1.209） | | 1.391 | （1.144，1.692） |
| 130 |  | 1.058 | （0.994，1.126） |  | 1.133 | （1.016，1.264） | | 1.473 | （1.158，1.873） |
| 140 |  | 1.069 | （0.992，1.152） |  | 1.163 | （1.020，1.326） |  | 1.562 | （1.170，2.084） |
| 150 |  | 1.081 | （0.990，1.180） |  | 1.195 | （1.024，1.394） | | 1.659 | （1.182，2.328） |
| 160 |  | 1.093 | （0.988，1.209） |  | 1.229 | （1.028，1.468） | | 1.763 | （1.192，2.606） |

Supplementary table 2-2. The overall cumulative association between preterm birth and PM_10_

|  | PM_10_ & 1-3 | |  | PM_10_ & 1-7 | |  | PM_10_ & 1-30 | |
| --- | --- | --- | --- | --- | --- | --- | --- | --- |
| Level (μg/m^3^) | RR | 95%CI |  | RR | 95%CI |  | RR | 95%CI |
| 20 | 0.993 | （0.961，1.026） |  | 1.005 | （0.950，1.064） |  | 0.919 | （0.827，1.021） |
| 30 | 0.994 | （0.970，1.019） |  | 1.003 | （0.961，1.046） | | 0.934 | （0.864，1.009） |
| 40 | 0.995 | （0.979，1.012） |  | 1.000 | （0.972，1.029） | | 0.949 | （0.901，1.001） |
| 50 | 0.997 | （0.988，1.006） |  | 0.999 | （0.983，1.016） | | 0.967 | （0.939，0.996） |
| 60 | 0.999 | （0.996，1.002） |  | 0.999 | （0.994，1.005） | | 0.987 | （0.977，0.997） |
| 70 | 1.001 | （0.999，1.003） |  | 1.001 | （0.998，1.004） | | 1.010 | （1.003，1.016） |
| 80 | 1.004 | （0.997，1.010） |  | 1.004 | （0.994，1.015） | | 1.036 | （1.016，1.056） |
| 90 | 1.007 | （0.997，1.016） |  | 1.009 | （0.993，1.026） | | 1.065 | （1.033，1.099） |
| 100 | 1.010 | （0.997，1.023） |  | 1.016 | （0.993，1.039） | | 1.099 | （1.052，1.147） |
| 110 | 1.014 | （0.997，1.031） |  | 1.024 | （0.995，1.053） | | 1.135 | （1.073，1.201） |
| 120 | 1.018 | （0.997，1.039） |  | 1.033 | （0.997，1.071） | | 1.176 | （1.093，1.265） |
| 130 | 1.023 | （0.997，1.049） |  | 1.044 | （0.999，1.091） | | 1.220 | （1.113，1.338） |
| 140 | 1.028 | （0.996，1.060） |  | 1.056 | （1.001，1.115） | | 1.268 | （1.131，1.422） |
| 150 | 1.033 | （0.994，1.072） |  | 1.069 | （1.001，1.142） | | 1.321 | （1.148，1.519） |
| 160 | 1.038 | （0.992，1.086） |  | 1.084 | （1.002，1.172） | | 1.377 | （1.163，1.630） |
| 170 | 1.044 | （0.989，1.101） |  | 1.099 | （1.001，1.206） | | 1.437 | （1.177，1.755） |
| 180 | 1.049 | （0.986，1.117） |  | 1.115 | （1.000，1.243） |  | 1.502 | （1.189，1.896） |
| 190 | 1.055 | （0.983，1.134） |  | 1.132 | （0.999，1.283） | | 1.571 | （1.201，2.055） |
| 200 | 1.062 | （0.979，1.151） |  | 1.150 | （0.998，1.326） | | 1.644 | （1.211，2.231） |
| 210 | 1.068 | （0.974，1.170） |  | 1.168 | （0.996，1.371） | | 1.721 | （1.221，2.427） |
| 220 | 1.074 | （0.970，1.189） |  | 1.188 | （0.993，1.420） |  | 1.803 | （1.230，2.642） |

Supplementary table 2-3. The overall cumulative association between preterm birth and SO_2_

| Level (μg/m^3^) | SO_2_ & 1-3 | |  | SO_2_ & 1-7 | |  | SO_2_ & 1-30 | |
| --- | --- | --- | --- | --- | --- | --- | --- | --- |
|  | RR | 95%CI |  | RR | 95%CI |  | RR | 95%CI |
| 5 | 0.966 | （0.933，1.001） |  | 0.944 | （0.886，1.005） | 0.780 | | （0.699，0.869） |
| 10 | 1.000 | （1.000，1.000） |  | 1.000 | （1.000，1.000） |  | 1.000 | （1.000，1.000） |
| 15 | 1.034 | （1.013，1.056） |  | 1.074 | （1.035，1.115） | 1.252 | | （1.174，1.335） |
| 20 | 1.069 | （1.023，1.117） |  | 1.170 | （1.082，1.266） | 1.531 | | （1.335，1.756） |
| 25 | 1.105 | （1.017，1.200） |  | 1.289 | （1.112，1.494） | 1.838 | | （1.418，2.382） |
| 30 | 1.141 | （0.996，1.308） |  | 1.430 | （1.120，1.825） |  | 2.176 | （1.419，3.336） |
| 35 | 1.179 | （0.966，1.438） |  | 1.595 | （1.116，2.281） | 2.554 | | （1.367，4.773） |

Supplementary table 2-4. The overall cumulative association between preterm birth and NO_2_

| Level (μg/m^3^) | NO_2_ & 1-3 | |  | NO_2_ & 1-7 | |  | NO_2_ & 1-30 | |
| --- | --- | --- | --- | --- | --- | --- | --- | --- |
|  | RR | 95%CI |  | RR | 95%CI |  | RR | 95%CI |
| 15 | 0.989 | （0.932，1.048） |  | 0.975 | （0.882，1.078） | | 0.975 | （0.807，1.177） |
| 20 | 0.990 | （0.948，1.034） |  | 0.978 | （0.909，1.053） | | 0.962 | （0.838，1.105） |
| 25 | 0.992 | （0.963，1.021） |  | 0.982 | （0.935，1.032） | | 0.955 | （0.871，1.047） |
| 30 | 0.994 | （0.977，1.010） |  | 0.987 | （0.959，1.015） | | 0.956 | （0.908，1.008） |
| 35 | 0.996 | （0.989，1.003） |  | 0.993 | （0.981，1.004） | | 0.970 | （0.949，0.991） |
| 40 | 1.000 | （1.000，1.000） |  | 1.000 | （1.000，1.000） |  | 1.000 | （1.000，1.000） |
| 45 | 1.005 | （0.999，1.010） |  | 1.009 | （1.000，1.018） |  | 1.050 | （1.033，1.068） |
| 50 | 1.010 | （0.999，1.021） |  | 1.020 | （1.001，1.039） | | 1.122 | （1.081，1.165） |
| 55 | 1.016 | （0.998，1.035） |  | 1.032 | （1.000，1.066） |  | 1.218 | （1.139，1.301） |
| 60 | 1.023 | （0.995，1.053） |  | 1.046 | （0.995，1.100） |  | 1.338 | （1.204，1.486） |
| 65 | 1.031 | （0.990，1.074） |  | 1.061 | （0.987，1.141） | | 1.485 | （1.276，1.728） |
| 70 | 1.039 | （0.983，1.097） |  | 1.077 | （0.976，1.188） | | 1.661 | （1.354，2.039） |
| 75 | 1.047 | （0.976，1.124） |  | 1.094 | （0.964，1.241） | | 1.869 | （1.437，2.430） |
| 80 | 1.056 | （0.968，1.152） |  | 1.111 | （0.951，1.298） | | 2.110 | （1.527，2.914） |

Supplementary table 2-5. The overall cumulative association between preterm birth and O_3_

| Level (μg/m^3^) | O_3_ & 1-3 | |  | O_3_ & 1-7 | |  | O_3_ & 1-30 | |
| --- | --- | --- | --- | --- | --- | --- | --- | --- |
|  | RR | 95%CI |  | RR | 95%CI |  | RR | 95%CI |
| 10 | 1.072 | （1.034，1.112） |  | 1.110 | （1.044，1.180） |  | 1.242 | （1.147，1.346） |
| 20 | 1.043 | （1.019，1.067） |  | 1.063 | （1.024，1.104） | | 1.136 | （1.083，1.191） |
| 30 | 1.018 | （1.008，1.028） |  | 1.025 | （1.008，1.043） | | 1.053 | （1.032，1.075） |
| 40 | 1.000 | （1.000，1.000） |  | 1.000 | （1.000，1.000） |  | 1.000 | （1.000，1.000） |
| 50 | 0.991 | （0.983，0.998） |  | 0.990 | （0.977，1.002） | | 0.979 | （0.966，0.993） |
| 60 | 0.990 | （0.976，1.003） |  | 0.993 | （0.970，1.016） |  | 0.988 | （0.963，1.013） |
| 70 | 0.996 | （0.977，1.015） |  | 1.009 | （0.976，1.043） | | 1.023 | （0.983，1.064） |
| 80 | 1.008 | （0.982，1.035） |  | 1.036 | （0.991，1.084） | | 1.084 | （1.021，1.152） |
| 90 | 1.027 | （0.992，1.062） |  | 1.075 | （1.014，1.140） |  | 1.173 | （1.074，1.281） |
| 100 | 1.050 | （1.005，1.097） |  | 1.124 | （1.042，1.212） | | 1.290 | （1.142，1.458） |
| 110 | 1.078 | （1.020，1.140） |  | 1.182 | （1.074，1.301） | | 1.439 | （1.224，1.690） |
| 120 | 1.110 | （1.036，1.189） |  | 1.250 | （1.110，1.407） |  | 1.621 | （1.321，1.988） |
| 130 | 1.145 | （1.054，1.244） |  | 1.326 | （1.150，1.529） |  | 1.840 | （1.433，2.362） |
| 140 | 1.183 | （1.073，1.304） |  | 1.410 | （1.192，1.667） | | 2.096 | （1.557，2.822） |

Supplementary table 2-6. The overall cumulative association between preterm birth and CO

| Level (μg/m^3^) | CO & 1-3 | |  | CO & 1-7 | |  | CO & 1-30 | |
| --- | --- | --- | --- | --- | --- | --- | --- | --- |
|  | RR | 95%CI |  | RR | 95%CI |  | RR | 95%CI |
| 500 | 0.974 | （0.933，1.018） | | 0.972 | （0.900，1.049） | | 0.833 | （0.729，0.952） |
| 1000 | 1.008 | （1.003，1.014） | | 1.014 | （1.005，1.023） | | 1.046 | （1.031，1.062） |
| 1500 | 1.085 | （1.037，1.134） | | 1.178 | （1.092，1.272） | | 1.464 | （1.250，1.715） |
| 2000 | 1.206 | （1.055，1.379） | | 1.503 | （1.190，1.899） | | 2.249 | （1.366，3.704） |
| 2500 | 1.372 | （1.053，1.786） | | 2.038 | （1.284，3.234） | | 3.676 | （1.377，9.812） |
| 2900 | 1.533 | （1.045，2.248） | | 2.661 | （1.360，5.206） | | 5.573 | （1.344，23.104） |


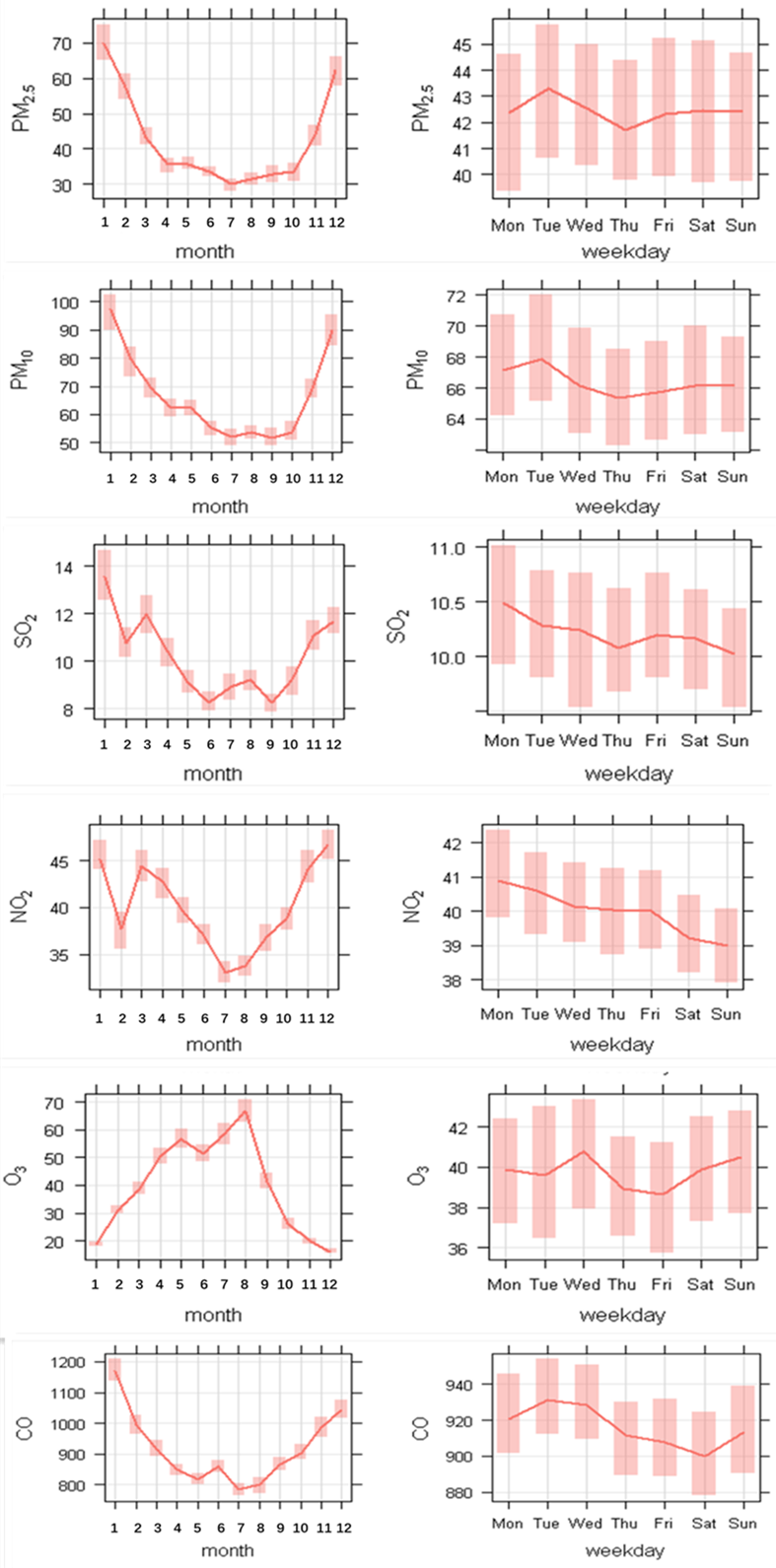


Supplementary fig.1. The time-series distribution of air pollutants in Chongqing, 2015-2020.
